# Practical Departments

**Published:** 1905-10-14

**Authors:** 


### PRACTICAL DEPARTMENTS.

### A MATERNITY BEDSTEAD.

Messrs. Monks, Hall and Co., of the Standard Bed Works, Warrington, have devised an admirable bedstead for maternity hospitals and private use. The bedstead itself is simply and strongly constructed with a good spring mattress, and guaranteed by the makers for fourteen years. Attached to the foot-posts of the bed, and between them, is a small cradle suspended hammock fashion. The advantage of such an arrangement in hospitals and in homes where space is limited is obvious. A cot or cradle at the side of the bed is very inconvenient, and ordinarily placed at the foot of the bed it is liable to escape observation, which is impossible if Monk's patent bedstead is used. The cot is, of course, detachable, and the bedstead available for ordinary use without it.

### MESSRS. EWART & SON'S GAS APPARATUS FOR HEATING WATER.

It is very often convenient to employ gas for heating purposes if it can be done with safety, especially for providing hot water for baths and lavatories, and Messrs. Ewart & Son, of 346-350 Euston Road, London, N.W., have worked out a complete system of what they call "Geysers," which they claim will accomplish what is required in this connection safely and at small cost. Their apparatus is divided into two classes—those in which the water is kept quite clear of the gas, and those in which the gas is allowed to pass through the water. The latter method is more economical, as the whole of the heat liberated by the combustion of the gas is delivered to the water. On the other hand there are many cases where this is not permissible, and for those the water is in tubes with the hot gases playing round them. In form the apparatus consists of a vertical cylinder, containing the water, and the passages for the hot gases, with provision for admitting the water supply, and underneath the water space a flat cylinder carrying a number of ordinary yellow flame burners, the burners being arranged to swing out clear of the water cylinder for lighting and cleaning. Ordinary yellow flame, or white flame, burners are used, because the blue Bunsen burner gives off noxious gases—carbonic oxide for instance. Messrs. Ewart point out that by arranging for the burners to swing out when lighting the danger of explosion is considerably lessened. They strongly recommend that when apparatus for heating water by gas is fitted in a bath-room there shall be a flue-pipe to carry off the fumes, leading into the open air, and they have designed a simple addition, to prevent down-draught where that is likely to take place, consisting of a system of baffles, fixed in a piece of pipe forming part of the flue. In addition some of the apparatus made by the firm are fitted with an automatic valve, by which the admission of gas is controlled by the admission of water. So long as water is flowing through the apparatus, being heated in the process, the gas remains on, but if the water stops the gas supply is cut off. The "Vulcan" valve, as they have called it, is very simple, and provided it is kept in order should preclude the accidents that have been made so much of, but, as Messrs. Ewart point out, have not been frequent where the gas has been extinguished, but the supply cock being left open, the gas has escaped into the room. With ordinary care this should never take place, and with the automatic appliance it should be impossible. In addition to the "Geysers" which are made by the firm in various forms and by various names, they also make boilers of different forms for quickly heating water for any purpose, also for circulating hot water for radiators; and they make a "Radiator" giving out heat by means of hot water, which is heated by gas, the heating apparatus being attached to and forming part of the radiator, the water being maintained continually in circulation within the radiator, and being re-heated as it passes through the heating-chamber after having given up its heat to the atmosphere of the room. The firm make various other appliances, such as the useful hot-towel rail and other accessories.

